# Fluorescence Imaging-Activated Microfluidic Particle Sorting Using Optical Tweezers

**DOI:** 10.3390/bios15080541

**Published:** 2025-08-18

**Authors:** Yiming Wang, Xinyue Dai, Qingtong Jiang, Hangtian Fan, Tong Li, Xiao Xia, Yipeng Dou, Yuxin Mao

**Affiliations:** School of Biomedical Engineering, Anhui Medical University, Hefei 230032, China; yimingwang@ahmu.edu.cn (Y.W.); 2213171090@stu.ahmu.edu.cn (X.D.); 2213170013@stu.ahmu.edu.cn (Q.J.); hangtianf@gmail.com (H.F.); 2445012554@stu.ahmu.edu.cn (T.L.); 2345012564@stu.ahmu.edu.cn (X.X.)

**Keywords:** fluorescence imaging, particle sorting, optical tweezers, microfluidics

## Abstract

The precise and efficient sorting of microscopic particles is critical in diverse fields, including biomedical diagnostics, drug development, and environmental monitoring. Fluorescence imaging-activated sorting refers to a strategy where fluorescence images are used to dynamically identify target particles and trigger selective manipulation for sorting purposes. In this study, we introduce a novel microfluidic particle sorting platform that combines optical tweezers with real-time fluorescence imaging for detection. High-speed image analysis enables accurate particle identification and classification, while the optical trap is selectively activated to redirect target particles. To validate the system’s performance, we used 10 µm green and orange fluorescent polystyrene particles. The platform achieved a sorting purity of 94.4% for orange particles under continuous flow conditions. The proposed platform provides an image-based sorting solution, advancing the development of microfluidic systems for high-resolution particle sorting in complex biological and environmental applications.

## 1. Introduction

The ability to sort microscopic particles is critical across a broad spectrum of scientific and industrial applications, including biomedical diagnostics, pharmaceutical development, environmental monitoring, and paleontological analysis [1,2,3]. Traditional techniques such as fluorescence-activated cell sorting (FACS) and magnetic-activated cell sorting (MACS) have long served as gold standards in particle separation. FACS utilizes fluorescence labeling and high-speed flow cytometry to identify and sort particles [4,5,6], whereas MACS isolates target populations via magnetic labeling and external magnetic fields [7,8,9]. However, despite their widespread use, both methods exhibit significant limitations. FACS systems are often bulky, expensive, and require complex fluidic handling and precise optical alignment, making them impractical for portable or point-of-care applications [10]. Meanwhile, MACS is constrained by its dependence on magnetic labeling, which is not universally compatible with all sample types and may introduce biological invasiveness [11].

To address these limitations, the past decade has witnessed growing interest in microfluidic-based particle sorting technologies [12]. Microfluidic platforms offer compelling advantages over conventional methods, including reduced sample/reagent consumption, compact device footprints, and enhanced functional integration with downstream analytical systems [13]. In recent years, various microfluidic cell sorting techniques have been developed, including acoustofluidic-based sorting [14], dielectrophoresis-based sorting [15], and hydrodynamic and inertial focusing methods [16]. Among the various microfluidic sorting strategies, optical tweezers have emerged as a particularly promising approach for non-invasive, high-precision microscale particle manipulation [17,18]. Optical tweezers exploit momentum transfer from a tightly focused laser beam to trap and manipulate dielectric particles in three dimensions, enabling contactless sorting with sub-micron spatial resolution [19,20,21]. When integrated into microfluidic channels, optical tweezers can selectively direct target particles into designated outlets by dynamically steering the trapping laser or by adjusting flow conditions [22].

On the other hand, effective particle separation critically depends on accurate target detection. Conventional approaches typically employ photomultiplier tubes (PMTs) or avalanche photodiodes (APDs) to measure fluorescence signals from labeled particles [23,24,25]. While these detectors provide excellent sensitivity and rapid response, they are fundamentally limited to single-dimensional signal acquisition and require meticulous optical alignment with the flow stream [26]. These limitations not only increase system complexity but also preclude the morphological characterization of sorted particles [27,28,29]. Recent advances in image-based detection are revolutionizing microfluidic sorting platforms [30,31,32,33]. High-speed cameras coupled with real-time image processing algorithms now enable comprehensive particle characterization, capturing detailed spatial and spectral information including size, shape, texture, and multi-color fluorescence signatures [34,35,36,37].

In this study, we present a novel microfluidic particle sorting platform that integrates optical tweezers with real-time image-based detection for the active separation of multi-color fluorescent particles. The imaging system precisely detects the color and gray value of each particle in the microchannel. These parameters trigger the targeted activation of the optical tweezers, enabling particle sorting via optical gradient forces. We validated the system performance through the continuous-flow sorting of orange and green fluorescent particles, demonstrating 94.4% sorting purity. This system holds promise for applications such as the precise sorting of different types of cells, screening fluorescently labeled microorganisms, and separating microplastic particles with distinct optical properties.

## 2. Materials and Methods

### 2.1. Working Principle of Fluorescence Imaging-Activated Microfluidic Particle Sorting System

**Figure 1:**
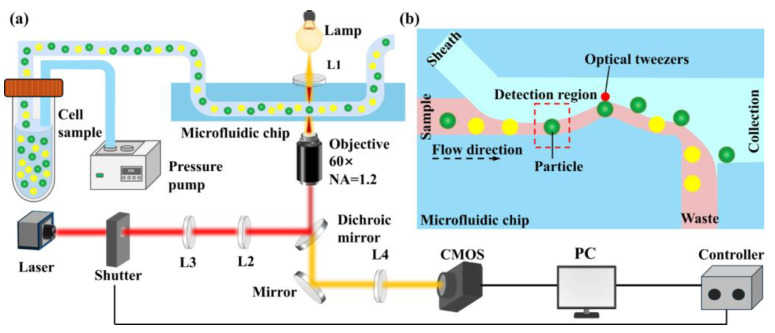
Working principle of fluorescence imaging-activated microfluidic particle sorting system. (**a**) System components. (**b**) Particle sorting process.

The sorting system integrates an image processing module into an optical tweezer particle sorting platform. As illustrated in Figure 1a, the system consists of a microfluidic module, an optical tweezer module, an imaging module, and a control software module. The entire setup is built around a microscope: the laser is emitted from a laser source, passes through a lens assembly, and is directed into the microscope. The microfluidic chip is placed on the microscope stage, while a high-speed camera is mounted on the microscope’s camera port for image acquisition. A computer located on a nearby table outside the optical platform is used for image capture and software-based processing.

Figure 1b shows a schematic diagram of the particle sorting process. As particles flow into the detection region, the high-speed camera captures their images. These images are processed in real-time using image processing software, which extracts the particle contours and subsequently derives brightness features. Based on these features, the system classifies the particle type and completes the identification process. Particle sorting is achieved by controlling the opening and closing of the laser shutter based on the image recognition results. During normal operation, the laser shutter remains closed. After an image is analyzed and a target particle is identified, the software opens the shutter to activate the optical tweezer at the sorting region, thereby capturing and sorting the target particle. If no target particle is detected, the shutter remains closed, allowing the particle to flow into the waste outlet.

### 2.2. Fabrication and Design of the Microfluidic Chip

The microfluidic chip consists of three layers. The bottom layer is a structural support layer, typically made of a microscope slide or coverslip, which enhances the overall rigidity of the chip. This layer is bonded to the side of the microfluidic structure containing the microchannels, ensuring a sealed environment. The middle layer is the main functional layer of the chip, which includes the sample inlet for the particle suspension, the sheath flow inlet for hydrodynamic focusing, the internal microchannel structures, and outlets for both the target and waste particles. The top layer serves as a connection interface, guiding the inflow and outflow of liquids. Since the inlet and outlet ports of the microfluidic chip often differ in size from the external tubing, this transition layer is essential for smooth fluid transfer into the chip.

The microfluidic chip is fabricated using a soft lithography technique. PDMS (SYLGARD 184, Dow, Midland, MI, USA) prepolymer and a curing agent are mixed at a 10:1 ratio and thoroughly stirred. The mixture is then degassed using a vacuum desiccator (PC-3, Shanghai Yueci Electronic Technology Co., Ltd., Shanghai, China) to remove air bubbles before being poured onto a silicon wafer mold. The mold is cured by heating at 70 °C for 1 h. After curing, the PDMS layer containing the microchannel structures is peeled off. To create access ports, a 1.6 mm diameter punch is used to form the sheath fluid inlets and outlets, while a 0.5 mm punch is used for the sample inlet. A separate 3 mm-thick PDMS slab is cast in a Petri dish and cut into appropriately sized blocks. A 1 mm diameter hole is punched in the center of each block to serve as the sample reservoir connection. Both the PDMS surface with the microchannels and the coverslip are treated with plasma (PDC-MG, Chengdu Mingheng Technology, Chengdu, China) for 90 s to activate the surfaces. They are then aligned and bonded together. The PDMS block for the sample reservoir is bonded to the chip using the same method. Finally, the assembled chip is heated at 80 °C for 1 h to enhance the bonding strength.

### 2.3. Image Acquisition

This system performs particle recognition through image-based detection. The image recognition program is developed in C++ using Visual Studio 2019. Image processing is carried out using the OpenCV computer vision library. The camera software provides a Software Development Kit (SDK), and additional functionalities are developed based on the basic image acquisition and display routines. Image data is acquired through the SDK and processed using OpenCV to extract the morphological features of particles, such as area, shape, and curvature, enabling effective particle identification. To verify the image recognition capability of the system, particle identification was performed under bright-field conditions, as illustrated in Supplementary Figure S1.

### 2.4. Optical Tweezer Setup

The optical tweezer setup consists of a laser source, shutter, lenses, and an objective lens. The laser source used is a 1064 nm laser (AFL-1064-37-R-CL, Amonics, Hongkong, China). A precision multifunctional timer (GCI-73, Daheng Optics, Beijing, China) controls the shutter placed immediately after the laser, offering a timing range from 1 ms to 2 h with a precision of 1 ms. Following the shutter is a polarizing beam splitter, which separates the laser beam into vertically and horizontally polarized components, allowing only one linearly polarized component to pass. Next, a half-wave plate is used to adjust the laser power by rotating its control knob, followed by a polarizer to further refine the beam. The laser beam is then redirected toward the microscope using three mirrors. Afterward, it passes through two lenses, one with a focal length of 150 mm and the other with a focal length of 250 mm, which serve to focus and collimate the beam. Finally, the beam is reflected by a dichroic mirror within the microscope into a 60× objective lens (Olympus, NA = 1.2). The objective focuses the laser onto the imaging plane, enabling the optical manipulation of particles.

Optical tweezers utilize the transfer of momentum from photons to dielectric particles, resulting in two primary components of optical force: the gradient force and the scattering force. The gradient force attracts particles toward the region of highest light intensity, typically the laser focus, while the scattering force pushes particles along the beam propagation direction. In our microfluidic setup, the gradient force dominates due to the high numerical aperture of the objective, enabling particles to be pulled from the sample fluid into the sheath fluid and directed into the collection channel. The laser power is carefully tuned to provide sufficient trapping strength (approximately 250 mW) while minimizing the risk of photodamage or thermal effects, ensuring compatibility with biological samples in future applications.

### 2.5. Selection of Shutter Opening Times

Precise control over the shutter opening timing is essential for stable particle sorting. The optical trap is set at a position 10 µm away from the microchannel ridge of the chip. The system uses a camera with a pixel size of 13.7 µm and a 60× objective lens. The particle size in the image can be calculated using the following formula:

(1) $$d=\frac{13.7}{60}\times N\;$$

where d is the particle size in micrometers and N is the number of pixels the particle occupies in the image. For a particle size of 10 µm, this corresponds to approximately 44 pixels.

To ensure reliable detection despite variations in particle velocity, the system defines three vertical monitoring points, spaced 15 pixels apart, starting from 30 pixels to the left of the center of the curved ridge. The shutter is triggered to open once a particle is detected at any of the monitoring points. Shutter actuation involves multiple communication steps—including signal transmission from the camera software (via a virtual serial port on the computer) to the laser shutter control software, and subsequently from the shutter control software to a high-precision timer via USB. Instead, repeated experimental tests were performed, and the optimal shutter opening time was determined to be 80 ms.

### 2.6. Sample Preparation

The preparation procedure for the particle solution is as follows: first, 1 mL of deionized water and 2 mL of human peripheral blood lymphocyte separation solution (Biosharp) (BL1420A, Beijing Lanjie Ke Technology Co., Ltd., Beijing, China)are drawn into a centrifuge tube and mixed thoroughly. Then, 10 µm of polystyrene particles (2.5% *w*/*v,* Jiangsu Zhichuan Technology Co., Ltd., Suzhou, China) is added to the mixture. To prevent particle aggregation, 0.03 g of the block copolymer Pluronic F-127 (P2443-250G, Sigma-Aldrich, Burlington, MA, USA) is also added to the solution.

## 3. Results

### 3.1. Particle Motion Under Sheath Flow

**Figure 2:**
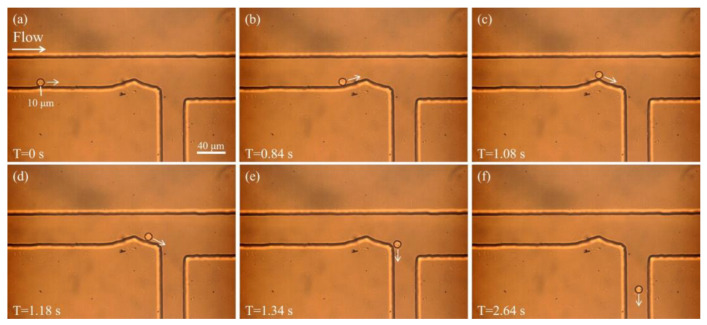
Particle motion under sheath flow. (**a**) The particle enters the main channel and is propelled in the direction of the fluid flow, as indicated by the arrow. (**b**–**d**) The particle continues to move along the edge of the curved channel, maintaining a stable trajectory near the lower side under the influence of the sheath flow. (**e**,**f**) After successfully navigating the bend, the particle continues along the lower wall and enters the lower outlet channel.

The formation of sheath flow is fundamental for effective particle sorting. When appropriate pressures are applied at both the sample and sheath inlets, the sheath fluid compresses the sample stream, causing the particles to migrate along the lower side of the microfluidic channel. Figure 2a illustrates the motion of a 10 μm particle under the influence of sheath flow. At T = 0 s, the particle enters the main channel and is propelled in the direction of the fluid flow, as indicated by the arrow. At this moment, the sheath fluid exerts pressure from the upper side, pushing the sample fluid and consequently the particle toward the lower wall of the channel. Figure 2b–d show the behavior of the particle as it passes through the curved section of the microfluidic channel. Over time, the particle continues to follow the edge of the curved path and maintains a stable trajectory near the lower boundary under the influence of the sheath flow. Figure 2e,f show the particle successfully navigating the bend and continuing to move along the lower wall without lateral deviation, eventually entering the lower outlet channel. This indicates that the sheath flow exerts precise control over the particle’s trajectory. These observations demonstrate that by adjusting the inlet pressures to establish a stable sheath flow, particles can be effectively guided along predetermined paths. This provides a solid foundation for achieving high-precision particle sorting downstream. 

### 3.2. Particle Motion Under Optical Tweezer Manipulation

**Figure 3:**
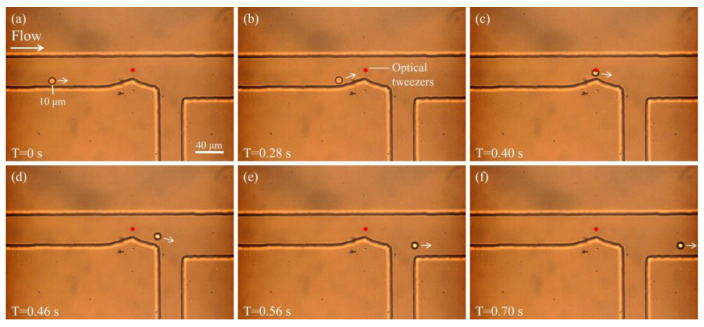
Particle motion under the combined influence of sheath flow and optical tweezers. (**a**) The particle enters the channel and moves along the lower wall. (**b**,**c**) As the particle approaches the optical tweezer, its trajectory begins to shift. (**d**–**f**) The particle is deflected from its original path by the optical tweezer and directed toward the upper outlet channel.

To verify the ability of optical tweezers to sort particles, a stationary optical tweezer was positioned at the bend of the microchannel. When a particle approaches the vicinity of the tweezer, its trajectory is altered due to the optical force. Figure 3 illustrates the motion of a 10 μm particle under the combined influence of sheath flow and optical tweezers. As shown in Figure 3a, at T = 0 s, the particle begins to move along the lower side of the microchannel driven by the sheath flow. At this time, the optical tweezer is already positioned above the bend (indicated by the red dot). Figure 3b,c show the particle gradually approaching the region influenced by the optical tweezer. At T = 0.40 s (Figure 3c), the particle enters the trap region and its trajectory begins to deflect due to the optical potential well. In Figure 3d–f, the particle is progressively “pushed” away from its original path near the lower wall and redirected toward the upper region of the channel. In Figure 3f, the particle clearly deviates from its initial trajectory and flows into the upper outlet. This result demonstrates that optical tweezers can exert a significant influence on particles in flow, effectively disrupting their stable sheath-aligned trajectories and enabling spatial control for sorting purposes.

### 3.3. Detection and Sorting of Fluorescent Particles

Accurate particle detection is fundamental for successful particle sorting. Upon acquiring the grayscale image captured by the camera, a region of interest (ROI) is first extracted to reduce computational load. We conducted particle sorting experiments using 10 µm orange and green fluorescent particles. The maximum excitation wavelength of the orange fluorescent particles is 532 nm, while that of the green fluorescent particles is 485 nm. Since both particles are identical in size (10 µm), they cannot be distinguished based on morphological features. Therefore, we differentiate them by analyzing their grayscale intensity values in fluorescence images.

**Figure 4:**
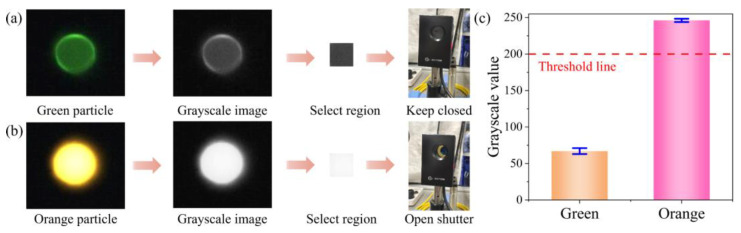
Detection of fluorescent particles with different colors. (**a**) Detection process of green particles. (**b**) Detection process of orange particles. (**c**) The grayscale value statistics of the selected region of fluorescent particles.

In the fluorescence images, the orange particles appear significantly brighter than the green ones. The difference in grayscale intensity is substantial, enabling classification based on the average pixel intensity. As shown in Figure 4a, we select a 30 × 30-pixel region located 30 pixels upstream of the microchannel ridge in the image captured by the camera. The average pixel intensity within this region is calculated using the grayscale fluorescence image. Results indicate that the average grayscale intensity of the orange fluorescent particles reaches approximately 246, whereas that of the green particles is only about 67 (Figure 4b). Based on this, a threshold of 200 is defined, as shown in Figure 4c. When the average pixel intensity exceeds this threshold, the particle is classified as an orange fluorescent particle, and the shutter is triggered to sort it accordingly.

**Figure 5:**
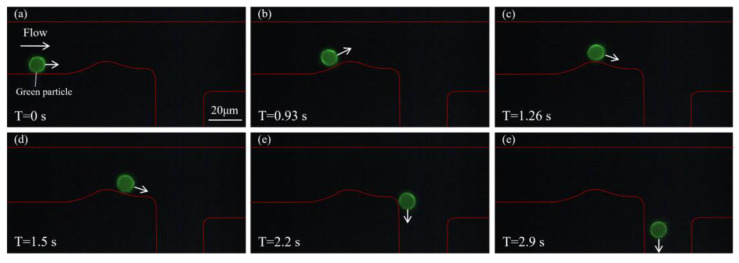
Sorting process of a green fluorescent particle. (**a**) At T = 0 s, the green particle enters the microfluidic channel and moves forward near the lower wall under the influence of sheath flow. (**b**,**c**) The particle continues to move steadily along the channel and approaches the bend, where it is identified by the detection system as a green particle, and the optical tweezer is not activated. (**d**,**e**) The green particle remains undisturbed and continues along the lower branch of the channel. (**f**) The particle is successfully discharged through the lower outlet.

To validate the system’s capability for particle sorting in a mixed environment, green and orange particles were mixed and subjected to sorting using the proposed microfluidic system. The initial concentration ratio of the two types of fluorescent particles was 1:1, as shown in Supplementary Figure S2. Figure 5 illustrates the sorting process of green particles. As shown in Figure 5a, at T = 0 s, a green particle enters the microfluidic channel and moves along the lower wall under the guidance of the sheath flow. The red outline indicates the channel boundary. As time progresses (Figure 5b), the particle continues along its predefined path and gradually approaches the curved region of the channel, where it is detected by the detection system. Upon identifying the particle as green, the optical tweezer remains inactive. In Figure 5c,d, the particle continues to travel smoothly along the lower side of the channel without disturbance. Furthermore, in Figure 5e,f, the green particle successfully navigates the bend, maintains a straight trajectory, and is eventually discharged through the lower outlet. The complete flow process of this green fluorescent particle is shown in Supplementary Video S1. This process demonstrates that the system effectively guides green particles along the designated path without interference from active manipulation tools.

**Figure 6:**
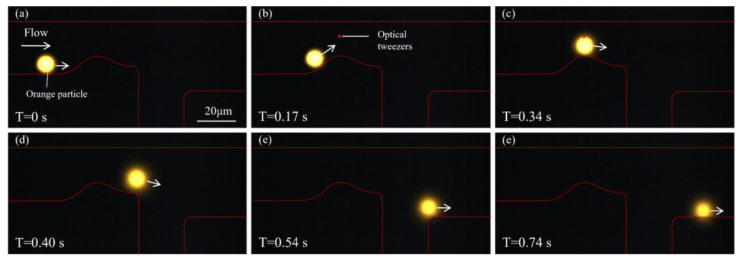
Sorting of an orange fluorescent particle. (**a**) At T = 0 s, the orange particle enters the microfluidic channel and moves along the lower wall under the guidance of sheath flow. (**b**,**c**) As the particle approaches the bend, it is identified by the detection system as an orange particle, triggering activation of the optical tweezer. The particle experiences an optical trapping force, causing its trajectory to begin shifting upward. (**d**,**e**) The particle gradually deviates from its original path and moves toward the upper region of the channel. (**f**) The particle is eventually discharged through the upper outlet.

Figure 6 illustrates the sorting process of an orange fluorescent particle. At T = 0 s, the orange particle enters the microfluidic channel and moves forward near the lower wall under the guidance of the sheath flow. The red outline indicates the channel boundary. As time progresses (Figure 6b), the particle continues along its predefined trajectory and gradually approaches the curved region of the channel, where it is detected by the system. Upon identifying the particle as orange, the optical tweezer is activated. In Figure 6c, as the particle enters the active region of the optical tweezer, its trajectory begins to deviate from the original path. Driven by the optical potential well, the particle is effectively pushed toward the upper part of the channel. In Figure 6d, a clear upward shift is observed, with the particle gradually leaving the original sheath-aligned path and moving toward the upper outlet. Figure 6e,f further show that the particle has completely deflected into the upper branch and is eventually discharged from the upper outlet, successfully separating its path from that of the green particles. The complete flow process of this orange fluorescent particle is shown in Supplementary Video S2. This process confirms the precise control of the particle trajectory, enabled by the optical tweezer for the target particles.

**Figure 7:**
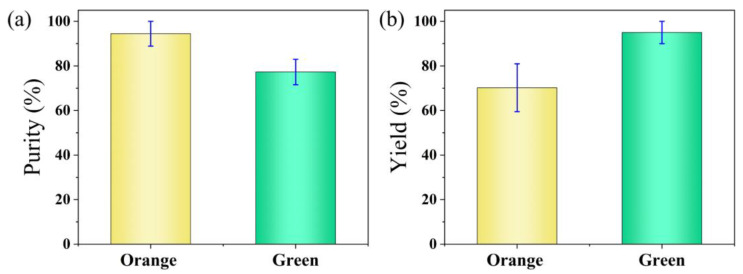
Statistical analysis of the sorting results for orange and green fluorescent particles. (**a**) Purity of the sorted orange and green fluorescent particles. (**b**) Yield of the sorted orange and green fluorescent particles.

Furthermore, we performed a statistical analysis of the sorting results by recording the number of orange and green particles flowing into the collection and waste outlets, respectively. Supplementary Video S3 demonstrates a continuous sorting process involving both types of particles. During the analysis, events involving adhesion between the two types of particles were excluded. A total of four datasets were collected. Figure 7a shows the purity of the two types of particles. The sorting purity of the orange fluorescent particles is defined as the proportion of orange particles at the collection outlet relative to the total number of orange and green particles at the collection outlet. The sorting purity of the green fluorescent particles is defined as the proportion of green particles at the waste outlet relative to the total number of orange and green particles at the waste outlet. The sorting purities of the orange and green particles were 94.4% and 77.3%, respectively. Figure 7b shows the yields of the two types of particles. The sorting yield of the orange fluorescent particles is defined as the proportion of orange particles at the collection outlet relative to the total number of orange particles at both the collection and waste outlets. The sorting yield of the green fluorescent particles is defined as the proportion of green particles at the waste outlet relative to the total number of green particles at both the collection and waste outlets. The sorting yields of the orange and green particles were 70.2% and 95.0%, respectively.

## 4. Discussion

In this study, we developed a particle sorting system by integrating optical tweezers with microfluidic image detection technology. However, there are still several notable limitations that need to be addressed in future work. First, the current work only performed sorting on two types of particles with different fluorescence colors, and the present design is suitable only for particles of approximately 10 µm in size. In the future, we plan to extend the system to sort more complex biological particle populations, such as cells at different stages of the cell cycle or extracellular vesicles (EVs). However, such complex particles exhibit significant heterogeneity in size, morphology, and fluorescence characteristics, which may affect detection accuracy and optical trapping efficiency. To address these challenges, future system upgrades may include adaptive laser power control, more advanced image classification algorithms, and the incorporation of multi-channel microfluidic architectures to enable parallel sorting. Second, the level of system integration should be improved. The current setup consists of multiple separate components and occupies considerable space. The assembly and disassembly of the equipment before and after experiments are both time-consuming and labor-intensive. A more practical solution would be to design a fully integrated instrument that consolidates all functional units into a compact device. This would reduce operational complexity and allow users to perform experiments simply by replacing the sample reservoir and microfluidic chip, thereby enhancing usability and efficiency. Finally, our system achieved an average sorting rate of 0.83 particles/min. Throughput could be further increased by raising the flow rate and particle concentration, as well as by reducing the detection time.

## 5. Conclusions

This study proposes a particle sorting technique based on an optical tweezer system combined with image-based recognition and validates its performance using polystyrene particles. The system captures particle images using high-speed imaging and classifies the particles based on these images. Once a target particle is identified, the software controls the opening and closing of the laser shutter, thereby enabling the precise formation of the optical trap and facilitating the sorting of the desired particle. A sorting test was performed using 10 µm green and orange fluorescent particles, achieving a sorting purity of 94.4% for the orange fluorescent particles. Our results highlight the potential of combining optical tweezing with image-based feedback to overcome the limitations of traditional and contemporary sorting techniques. This work not only contributes to a practical solution for high-resolution particle sorting, but also lays the groundwork for future developments in intelligent, adaptive microfluidic systems capable of handling more complex sorting tasks, including those involving biological particles or heterogeneous environmental samples.

## Data Availability

The data are available from the corresponding author upon reasonable request.

## Supplementary Materials

The following supporting information can be downloaded at: https://www.mdpi.com/article/10.3390/bios15080541/s1. Figure S1: Detection process of bright-field particle images. Figure S2: Fluorescence images of the two types of particles before sorting. Video S1: Flow process of a green fluorescent particle. Video S2: Flow process of an orange fluorescent particle. Video S3: Continuous sorting process of green and orange fluorescent particles
