# Supplementary material for: Fluorescence Imaging-Activated Microfluidic Particle Sorting Using Optical Tweezers
Source: Biosensors (Basel). 2025 Aug 18;15(8):541. doi: 10.3390/bios15080541 (PMC12384192; doi:10.3390/bios15080541)
Supplement: Supplementary file 1 [file biosensors-15-00541-s001.zip › Supplementary material Figures S1 and S2.zip!/Supplementary material Figures S1 and S2.pdf]

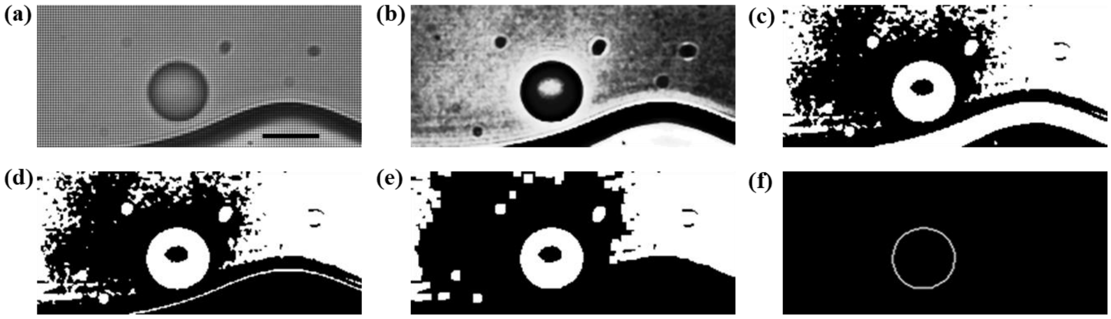


Figure S1. Detection process of bright-field particle images. (a) Original image. (b) Histogram equalization. (c) Otsu binarization. (d) Removal of microfluidic chip components. (e) Erosion and dilation processing. (f) Particle contour extraction. The scale bar is 10 μm.

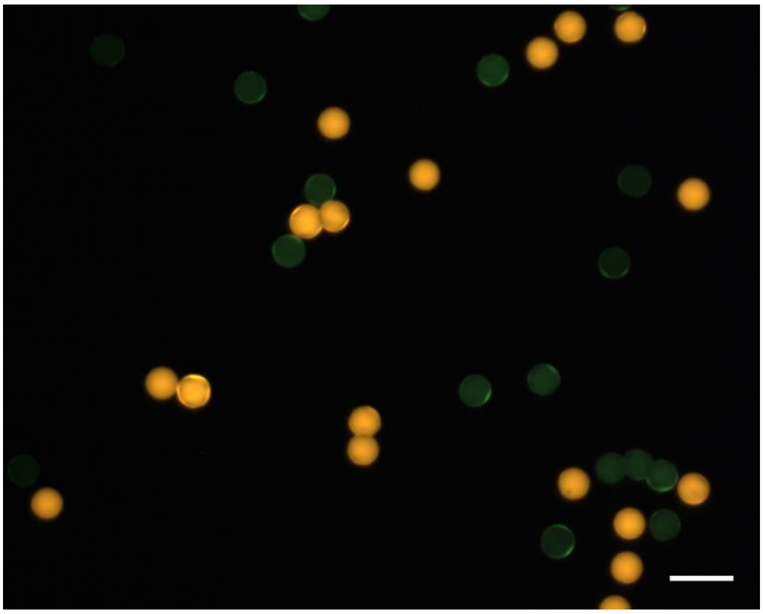

Figure S2. Fluorescence images of the two types of particles before sorting. The scale bar is 20 μm.
